# The Effect of Social Support on Athlete Burnout in Weightlifters: The Mediation Effect of Mental Toughness and Sports Motivation

**DOI:** 10.3389/fpsyg.2021.649677

**Published:** 2021-05-14

**Authors:** Yao Shang, Shi-Yong Yang

**Affiliations:** Faculty of Athletics and Swimming, Chengdu Sports University, Chengdu, China

**Keywords:** social support, mental toughness, sports motivation, athlete burnout, weightlifter

## Abstract

**Objectives:** Athlete burnout is a crucial concern affecting the development and athletic performance of young weightlifters. To reduce or relieve the prevalence of athlete burnout, this study examined the relationship across social support, sports motivation, mental toughness, and athlete burnout in weightlifters.

**Methods:** A total of 315 weightlifters aged 17–28 years old (151 males, 164 females; *M*_*age*_ = 18.89 years, *SD* = 3.66) from Sichuan, Chongqing, and Shanxi in China participated in this survey. The Perceived Available Support in Sport Questionnaire (PASS-Q), Sports Motivation Questionnaire (SMQ), Sports Mental Toughness Questionnaire (SMTQ), and Athlete Burnout Questionnaire (ABQ) were used in this study. SPSS Statistics 19.0, AMOS 21.0, and PROCESS 3.0 macro were used to analyze the collected data.

**Results:** The results indicated that weightlifters’ social support could negatively significantly affect athlete burnout [beta = −0.398; 95% confidence interval (CI): −0.3699, −0.2184; *P* < 0.05) *via* mental toughness and sports motivation. The mediation analysis revealed that they had partial mediating effect, including three paths: First, social support had a direct effect on athlete burnout (beta = −0.150; 95% CI: −0.1824, −0.0397; *P* < 0.05); second, sport mental toughness had a mediating effect on athlete burnout (beta = −0.113; 95% CI: −0.1703, −0.0631; *P* < 0.05); and finally, sports motivation had a mediating effect on athlete burnout (beta = −0.124; 95% CI: −0.1751, −0.0793; *P* < 0.05).

**Conclusion:** The findings revealed that social support could inhibit or prevent athlete burnout *via* mental toughness and sports motivation; thus, to decrease or relieve the prevalence of burnout in weightlifters, it is an important solution to enhance their social support.

## Introduction

Weightlifting needs performers’ skilled techniques, strong physical performance, and excellent psychological characteristics to exert their maximum efforts to lift optimal weights (Dvorkin, 2005). Nowadays, the performance gap among the world’s top weightlifters has gradually narrowed. Thus, the role of psychological quality, which can be measured by scales on emotion, confidence, willpower, resilience, and so on, is becoming more and more critical. A variety of studies have shown that when competing with opponents with equal ability, the athletes’ psychological differences, such as toughness, cognition, or personality, can affect the competition outcomes by more than 50% (Weinberg and Gould, 2003). Therefore, excellent psychological quality has become an essential factor in athletes’ evaluation (Chang et al., 2012; Lou et al., 2014). Thus, monitoring the psychological state of athletes has an important practical significance.

Athlete burnout has received widespread attention due to its negative impact on athletes’ training and competition (Chen and Zhou, 2007; Goodger et al., 2007). This term was originally proposed by Freudenberger (1974). It is used to describe the exhaustion of psychological resources in long-term and high-stress environments, mostly in high-stress industries such as doctors and nurses. The research on burnout in the field of sports psychology started in the 1980s. Weinberg and Gould (2003) believed that athlete burnout is a response to overtraining (both psychological and physiological stress), which is a type of athlete’s difficulty in maintaining regular training and reaching the optimal state of the past performance. To effectively distinguish it from physiological fatigue and fully demonstrate its development process, Zhang et al. (2006) defined it as “mental fatigue,” which mainly refers to the comprehensive performance of the decline in psychological function caused by athletes’ failure to replenish psychological and physiological resources in the process of continuous consumption when coping with pressure. Hu and Ning (2015) pointed out that professional weightlifting training will cause deep fatigue in the central nervous system, musculoskeletal system, and respiratory system of weightlifters, and it will take a long time to recover. The production of mental fatigue not only damages the athletes’ mental health but also may cause their withdrawal from training. As a result, many scholars are committing to find out some effective approaches to relieve athletes’ mental burnout.

The occurrence and development of athlete burnout is related to a series of physical, psychological, and sociological factors, among which social support and mental toughness are two very important protective factors (Lou et al., 2014; Ye et al., 2016). However, there are few models that emphasize the interaction between personality and environmental aspects (Goodger et al., 2007). In addition, whether it is the cognitive-affective model proposed by Smith (1986), the commitment model of Raedeke (1997), or the athlete burnout integration model proposed by Gustafsson et al. (2011), the decline of sports motivation all plays an important role in the production of athlete burnout. Gustafsson et al. (2011) believed that the diminishing motivation is an early symptom of athlete burnout. Through literature review and analysis, it was found out that mental toughness and motivation may play a mediating role between the effect of social support on athlete burnout.

Social support commonly refers to the various forms of support and assistance provided by the individual’s social network system, such as care, attention, or respect from other members in the social network, which directly affect the individual’s health (Uchino et al., 1996). The stress model believes that any factors related to increased stress levels may cause mental burnout (Zhang et al., 2014). Previous studies have shown that social support can “buffer” stress through spiritual and material comfort, care, respect, and help from family, relatives, and other members of the society (Fisher, 1985; Kaiseler et al., 2009). It is not difficult to speculate that more social support can help athletes reduce fatigue in stressful situations. The research by Zhang and Zhi (2018) also suggested giving athletes more support to regulate the production of mental fatigue.

Mental toughness, also known as psychological resilience, refers to the process of an individual’s good adaptation in the face of adversity, trauma, or stress (White et al., 2008). Jones et al. (2002) found out that many studies exploring the causes of successful athletes used mental toughness as an essential component, and their findings pointed out that mental toughness is an inherited or acquired psychological advantage, enabling athletes to better cope with stress in competition, training, and life than their counterparts (Jones et al., 2007). According to the stress model explaining the induction of mental fatigue (Zhang et al., 2014), it is not difficult to speculate that mental toughness can inhibit mental fatigue. The mentally tough athletes can positively evaluate external stimuli in stressful situations, effectively control and regulate their own emotions, and possess more optimistic expectations for goal pursuit (Madigan and Nicholls, 2017). These positive emotions and behavioral tendencies can protect athletes from sports fatigue symptoms. On the other hand, mental toughness is not static. In addition to genetic factors, the influence of the acquired environment is equally important. Previous studies believed that social support results in important attributions to mental resilience (Jones et al., 2002). Connaughton et al. (2010) also suggested that the formation of a good social support network around athletes has important practical significance for improving mental toughness.

Motivation is the psychological motivation or internal driving force that promotes a person’s activities, and it has the initiating, directing, and strengthening effect on people’s behaviors. Sports motivation is the internal motivation that causes and maintains individuals to engage in sports and to strive to reach their goals (Ma and Zhang, 2003). Sports motivation directly affects the enthusiasm and investment of athletes in training and competition. It then affects their training effect, competition performance, and career development (Zhang, 2015). Previous studies have investigated widely the correlation between sports motivation and mental burnout (Gould et al., 1996; Goodger et al., 2007). For example, Zhang et al. (2010) found that internal motivation and mental burnout were negatively correlated, while the lack of motivation was positively correlated with mental burnout. Besides, Harter’s (1987) behavioral motivation theory and the overall self-worth mediation model believed that in sports, society, and many other fields, the support of coaches, parents, and peers contributes to individual self-worth perception, which can directly and indirectly affect motivation and related behaviors.

Many studies have shown that athletes’ mental toughness is closely related to their motivation. Zeng and Liu (2013) found out that the scores of athletes’ sports motivation and participation tendency are significantly correlated with mental toughness dimensions. Strümpfer (2003) pointed out that the emergence of mental toughness can promote individual fitness, which leads to a trend opposite to the tendency of burnout, and athletes with higher levels of mental toughness are more motivated and show better persistence in goal pursuit behavior.

In short, social support, mental toughness, and sports motivation can alleviate the generation of athlete burnout, while social support can affect mental toughness; social support and mental toughness can also affect sports motivation. It can be seen that mental toughness and sports motivation may be the mediators of social support on athlete burnout. However, previous studies have mostly used social support as the unimoderator (Zhou and Guo, 2007; Wang, 2013) and paid little attention to its mechanism of athlete burnout. Based on this, the present study proposed the following four hypotheses to examine the impact of weightlifters’ social support on athlete burnout and the role of mental toughness and sports motivation, clarify the value of social support, enrich the path of social support affecting athlete burnout, to provide theoretical bases for further explaining the mechanism of social support on athlete burnout, and provide ideas for the prevention and intervention of athlete burnout.

Hypothesis I: The social support of weightlifters can negatively affect athlete burnout.
Hypothesis II: The mental toughness of weightlifters plays a mediating effect between social support and athlete burnout.
Hypothesis III: The sport motivation of weightlifters plays a mediating effect between social support and athlete burnout.
Hypothesis IV: The mental toughness and sport motivation of weightlifters play a serial mediating effect across social support and athlete burnout.

## Materials and Methods

### Participants

In this study, a sample of 327 weightlifters from Sichuan, Chongqing, and Shanxi in China was recruited as participants by communicating with their head coaches *via* email or telephone. Each participant filled out the survey questionnaire online according to the instruction of the scale and their coaches during December 12, 2019, to January 21, 2020. After completing the survey, the data preparation was carried out. Twelve copies were found to have incomplete or missing data; 315 copies were valid. The valid rate was 96.3%. The ages of the participants ranged from 17 to 28 years old (*M* = 18.89; *SD* = 3.66). For more information on the participants, see Table 1.

**TABLE 1:** Summary of the sociodemographic information of the respondents.

**Category**		**Frequency**	**Percent**
Gender			
	Female	151	47.9
	Male	164	52.1
Athletic level*			
	Master sportsman	45	14.3
	Level 1	70	22.2
	Level 2	200	63.5
Origin			
	Rural	279	88.6
	Urban	36	11.4
Education level			
	College	144	45.7
	High school	96	30.5
	Others	75	23.8
Household income (yuan/month)			
	Less than 2,000	66	21
	2,000–4,000	161	51.1
	4,000–6,000	51	16.2
	6,000–8,000	22	7
	8,000–10,000	9	2.9
	10,000+	6	1.9
Training experience (years)			
	Less than 2	105	33.3
	2–5	78	24.8
	5+	132	51.9

*Based on the criteria of the Chinese State General Administration of Sports.

This study was approved by the Ethics Committee of Chengdu Sports University following the Declaration of Helsinki. Each participant provided written informed consent, and the warranty of confidentiality was promised to them.

### Instruments

#### Perceived Available Support in Sport Questionnaire

The Perceived Available Support in Sport Questionnaire (PASSQ) developed by Freeman et al. (2011) was used to test the social support of the respondents. The questionnaire has 16 items, including four dimensions: emotional support, e.g., “provide you with comfort and security”; esteem support, e.g., “instill you with the confidence to deal with pressure”; informational support, e.g., “give you constructive criticism”; and tangible support, e.g., “help with travel to training and matches.” A five-point Likert-type scale ranging from 1 (never) to 5 (always) was used. The total score of social support is the sum of the scores of 16 items. A higher score represents a higher perceived level of social support.

#### Athlete Burnout Questionnaire

The Athlete Burnout Questionnaire (ABQ) developed by Raedeke and Smith (2001) was used to assess the level of mental burnout of weightlifters in this study. There are 15 items organized into three dimensions in this inventory: physical and emotional exhaustion (PEE), which contains five items, e.g., “I feel extremely tired from the sport participation” and “I feel physically and emotionally worn out by sport”; reduced sense of achievement (RSA), which contains five items, e.g., “I feel successful at sports” and “It seems that no matter what I do, I don’t perform as well as I should!”; and devaluation of sports practice (DSP), which contains five items, e.g., “I am not as worried about being successful as I used to be” and “I am not as interested in sport as I used to be.” The five-point Likert-type scale from 1 (never) to 5 (always) is used. The first and 14th items are reverse-coded items. The total score of the ABQ is the sum of all 15 items. The higher the score, the higher the burnout level of the weightlifters.

#### Sports Mental Toughness Questionnaire

The Sports Mental Toughness Questionnaire (SMTQ) compiled by Sheard et al. (2009) and revised by Wang et al. (2014) was used to test the mental toughness of weightlifters. It contains a total of 12 items with three dimensions: confidence, e.g., “I will set challenging goals for myself”; constancy, e.g., “Having an insatiable desire and internalized motives to succeed”; and control, e.g., “I will become anxious because things cannot be predicted or controlled.” A five-point Likert-type scale ranging from 1 (completely inconsistent) to 5 (completely consistent) was used. The total score of the mental toughness was the sum of all 12 items. The higher the score, the stronger the mental toughness.

#### Sports Motivation Questionnaire

The Sports Motivation Questionnaire (SMQ) developed by Zhang and Mao (2004) was used to test the respondents’ sports motivation. The questionnaire contains six items, consisting of two dimensions: approach tendency, e.g., “I can get a lot of fun from the sports I am engaged in”; and avoidance tendency, e.g., “If I can choose, I will practice other sports.” A five-point Likert-type scale from 1 (completely disagree) to 5 (completely agree) was used. The higher the score, the stronger the motivation to exercise. The following formula obtained the total score: score of item 1 + score of item 3 + score of item 5 − score of item 2 − score of item 4 − score of item 6 + 18.

### The Reliability and Validity Measures of the Scales

As shown in Table 2, the Kaiser–Meyer–Olkin (KMO) value of the PASSQ was 0.79 (*P* > 0.5). The Bartlett sphere test was less than 0.05. These showed that they were suitable for factor analysis. The percentage of cumulative variance explained of PASSQ was 77.99%, i.e., the four dimensions of emotional, esteem, informational, and tangible accounted for 19.10, 23.14, 15.10, and 20.65%, respectively. The internal consistency Cronbach’s α coefficients were 0.91, 0.92, 0.84, and 0.90, respectively, indicating that the PASSQ has good reliability. The composite reliability (CR) of the four subscales were 0.91, 0.92, 0.84, and 0.90, respectively, and the average variance extraction (AVE) values were 0.72, 0.74, 0.57, and 0.70, respectively, indicating that the convergent validity of the scale is good.

**TABLE 2:** Reliability and validity test of the questionnaire.

**Construct**	**KMO and Bartlett’s test**	**Items**	**% variance explained**	**% cumulative variance explained**	**Cronbach’s α**
Emotional	0.79 *P* < 0.01	4	19.10	19.10	0.91
Esteem		4	23.14	42.24	0.92
Informational		4	15.10	57.34	0.84
Tangible		4	20.65	77.99	0.90
Physical and emotional exhaustion	0.84 *P* < 0.01	5	16	16	0.79
Reduced sense of achievement		5	12.97	28.97	0.72
Devaluation of sports practice		5	25.86	54.83	0.79
Confidence	0.76 *P* < 0.01	5	24.53	24.53	0.73
Constancy		3	16.61	41.14	0.74
Control		4	21.37	62.51	0.77
Approach tendency	0.69 *P* < 0.01	3	45.30	45.30	0.83
Avoidance tendency		3	25.62	70.92	0.67

The ABQ’s KMO value was 0.84, and the Bartlett sphere test result was less than 0.05. The percentage of cumulative variance explained of ABQ was 54.83%. Cronbach’s α coefficients of the three dimensions of emotional and physical exhaustion, reduced sense of achievement, and devaluation of sports practice were 0.79, 0.72, and 0.79, respectively; CR values were 0.79, 0.54, and 0.80, respectively; and AVE values were 0.45, 0.17, and 0.46, respectively.

The SMTQ’s KMO value was 0.76, and the Bartlett sphere test result was less than 0.05. The percentage of cumulative variance explained of MTQ was 62.51%. Cronbach’s α coefficients of the three dimensions of confidence, constancy, and control were 0.73, 0.74, and 0.77, respectively; CR values were 0.71, 0.86, and 0.78, respectively; and AVE values were 0.37, 0.82, and 0.48, respectively.

The SMQ’s KMO statistical value was 0.69, and the Bartlett sphere test result was less than 0.05. The percentage of cumulative variance explained of SMQ was 70.92%. Cronbach’s α coefficients of the two dimensions of approach tendency and avoidance tendency were 0.83 and 0.67, respectively; CR values were 0.85 and 0.71, respectively; and AVE values were 0.65 and 0.46, respectively.

### Statistical Analyses

SPSS Statistics 19.0, AMOS 21.0, and PROCESS 3.0 macro program (Hayes, 2018) were used to process and analyze the data. Descriptive statistics such as frequency analysis, exploratory factor analysis (EFA), confirmatory factor analysis (CFA), regression analysis, and mediation analysis were used in this study. The significance level of all variables was set to α = 0.05.

## Results

### Correlation Analysis of Weightlifters’ Social Support, Sports Motivation, Mental Toughness, and Athlete Burnout

As shown in Table 3, the three dimensions of emotional support, esteem support, and informational support in the social support scale were negatively significantly associated with athlete burnout (*R* = −0.23 ∼ −0.52). All dimensions of the sports motivation scale were negatively associated with each dimension of athlete burnout (*R* = 0.24 ∼ 0.44). The confidence dimension in the mental toughness scale was negatively associated with reduced sense of accomplishment (*R* = −0.43) and emotional and physical exhaustion (*R* = −0.36). The constancy dimension was negatively associated with all dimensions of athlete burnout (*R* = −0.34 ∼ −0.49). The control dimension was negatively associated with emotional and physical exhaustion (*R* = −0.33). Besides, there were significant correlations between social support, sports motivation, and mental toughness. The significant correlations provide better foundation for subsequent research hypotheses and mediation testing.

**TABLE 3:** Descriptive statistics and correlation coefficients between social support, sports motivation, mental toughness, and athlete burnout.

		***M* (*SD*)**	**1**	**2**	**3**	**4**	**5**	**6**	**7**	**8**	**9**	**10**	**11**	**12**
(1)	Emotional support	15.36 (3.88)	1											
(2)	Esteem support	14.72 (3.92)	0.72**	1										
(3)	Informational support	14.88 (3.25)	0.52**	0.53**	1									
(4)	Tangible support	16.74 (3.42)	0.25**	0.20*	0.54**	1								
(5)	Approach tendency	12.75 (2.06)	0.20*	0.29**	0.09	0.07	1							
(6)	Avoidance tendency	6.77 (3.01)	−0.28**	−0.27**	−0.23*	−0.29**	−0.23*	1						
(7)	Confidence	15.46 (3.61)	0.28**	0.39**	0.17	−0.04	0.17	−0.04	1					
(8)	Constancy	10.42 (2.02)	0.39**	0.47**	0.19	0.02	0.29**	−0.17	0.64**	1				
(9)	Control	12.44 (3.38)	0.17	0.21*	0.001	−0.17	0.03	−0.11	−0.04	0.12	1			
(10)	Reduced sense of accomplishment	12.63 (2.58)	−0.29**	−0.38**	−0.23*	−0.11	−0.27**	0.24*	−0.43**	−0.39**	−0.11	1		
(11)	Emotional/physical exhaustion	11.31 (3.46)	−0.34**	−0.52**	−0.26**	−0.03	−0.32**	0.35**	−0.36**	−0.49**	−0.33**	0.54**	1	
(12)	Devaluation of sports practice	9.91 (3.65)	−0.31**	−0.37**	−0.24*	−0.06	−0.30**	0.44**	−0.15	−0.34**	−0.18	0.49**	0.73**	1

M, mean; SD, standard deviation. *P < 0.05, **P < 0.01.

### The Regression Analysis of Social Support, Sports Motivation, Mental Toughness, and Athlete Burnout in Weightlifters

**FIGURE 1:**
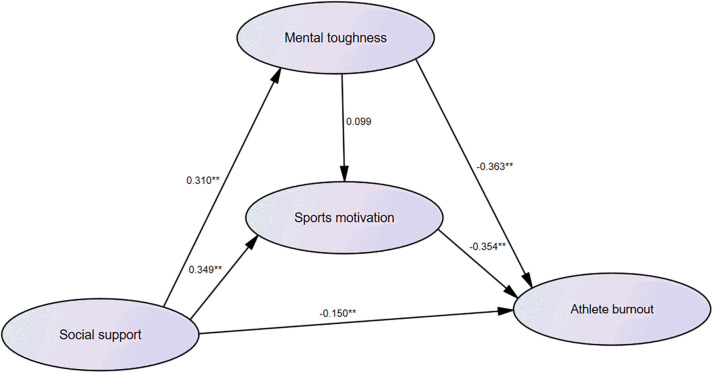
Schematic diagram of path analysis of social support, sports motivation, mental toughness, and athlete burnout. **P < 0.01.

Table 4 shows the following results: First, with social support as the independent variable and mental toughness as the dependent variable, the regression coefficient was statistically significant (beta = 0.310, *P* < 0.01), indicating that social support significantly affected mental toughness. With social support and mental toughness as the independent variables and sports motivation as the dependent variable, the regression coefficient of social support was statistically significant (beta = 0.349, *P* < 0.0l), but the regression coefficient of mental toughness was not statistically significant (beta = 0.099, *P* > 0.05). It showed that social support had a significant effect on sports motivation, but mental toughness had no significant impact on sports motivation. Taking social support, mental toughness, and sports motivation as independent variables and athlete burnout as the dependent variable, the regression coefficients of social support (beta = −0.150, *P* < 0.01), mental toughness (beta = −0.363, *P* < 0.01), and sports motivation (beta = −0.354, *P* < 0.01) were statistically significant, indicating that social support, mental toughness, and sports motivation all had a significant impact on the burnout of athletes (for the relationship, see Figure 1).

**TABLE 4:** Regression analysis of social support, sports motivation, mental toughness, and athlete burnout in weightlifters.

	**Social support**		**Mental toughness**		**Sports motivation**		***R*^2^**
	**β**	***T***	**β**	***T***	**β**	***T***	
Mental toughness	0.310	5.77**					0.096
Sports motivation	0.349	6.37**	0.099	1.8			0.153
Athlete burnout	−0.150	−3.06**	−0.363	−7.85**	−0.354	−7.45**	0.406
RSA	−0.145	−0.27**	−0.344	−6.67**	−0.195	−3.37**	0.258
PEE	−0.130	−2.65**	−0.442	−9.57**	−0.282	−5.93**	0.406
DSP	−0.116	−2.15*	−0.166	−3.25**	−0.403	−7.69**	0.278

RSA, reduced sense of accomplishment; PEE, physical and emotional exhaustion; DSP, devaluation of sports practice. *P < 0.05, **P < 0.01.

**FIGURE 2:**
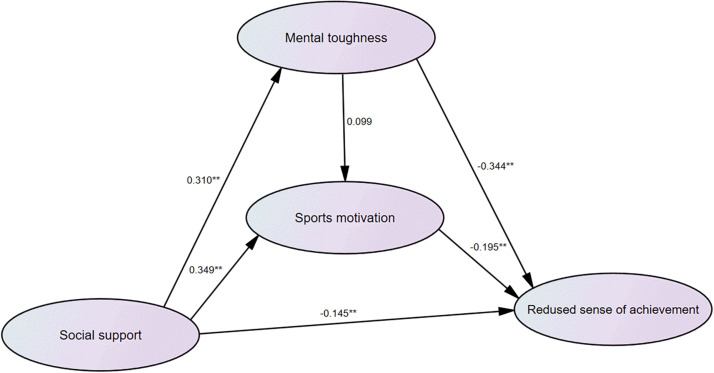
Schematic diagram of path analysis of social support, sports motivation, mental toughness, and reduced sense of achievement. **P < 0.01.

Second, with social support, mental toughness, and sports motivation as independent variables and reduced sense of achievement as the dependent variable, the regression coefficients of social support (beta = −0.145, *P* < 0.01), mental toughness (beta = −0.344, *P* < 0.01), and sports motivation (beta = −0.195, *P* < 0.01) were statistically significant, indicating that social support, mental toughness, and sports motivation all had a significant impact on the subdimension of athlete burnout (decreased sense of accomplishment). The regression weights of path analysis are listed in Figure 2.

**FIGURE 3:**
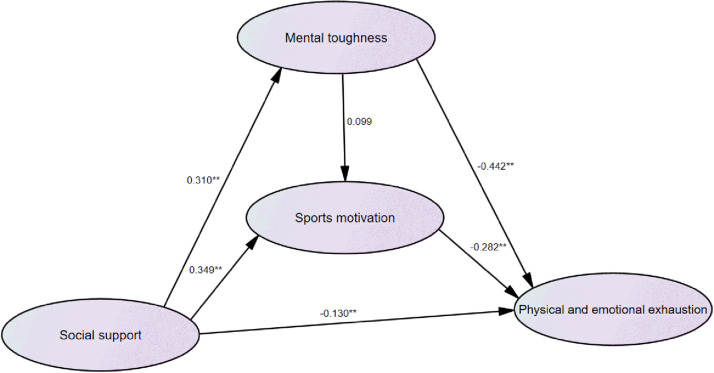
Schematic diagram of path analysis of social support, sports motivation, mental toughness, and physical and emotional exhaustion. **P < 0.01.

Third, with social support, mental toughness, and sports motivation as independent variables and physical and emotional exhaustion as the dependent variable, the regression coefficients of social support (beta = −0.130, *P* < 0.01), mental toughness (beta = −0.442, *P* < 0.01), and sports motivation (beta = −0.282, *P* < 0.01) were statistically significant, indicating that social support, mental toughness, and sports motivation all had a significant impact on the subdimension of athlete burnout (physical and emotional exhaustion). The regression weights of path analysis are listed in Figure 3.

**FIGURE 4:**
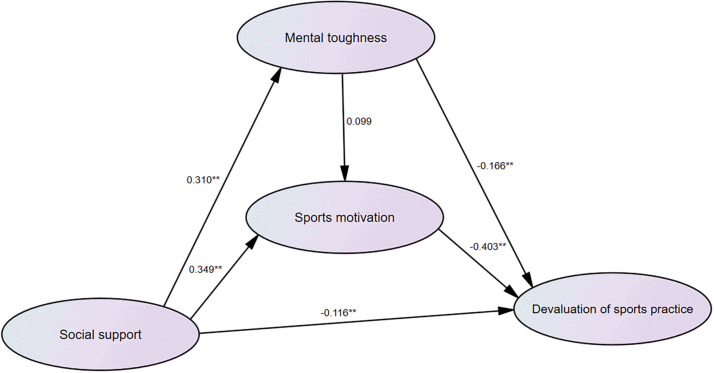
Schematic diagram of path analysis of social support, sports motivation, mental toughness, and devaluation of sports practice. **P < 0.01.

Fourth, with social support, mental toughness, and sports motivation as independent variables and devaluation of sports practice as the dependent variable, the regression coefficients of social support (beta = −0.116, *P* < 0.01), mental toughness (beta = −0.166, *P* < 0.01), and sports motivation (beta = −0.403, *P* < 0.01) were statistically significant, indicating that social support, mental toughness, and sports motivation all had a significant impact on the subdimension of athlete burnout (devaluation of sports practice). The regression weights of path analysis are listed in Figure 4.

As shown in Table 5, SMT played a mediating role between PASS and AB (indirect effect = −0.113; 95% CI: −0.1703, −0.0631). Also, SM played a mediating role between PASS and AB, and the mediating effect was −0.124 (95% CI: −0.1751, −0.0793); SMT had no significant effect on SM, which led to the failure of the chain mediating effect of SMT and SM between PASS and AB (95% CI: −0.0269, 0.0037). SMT played a mediating role between PASS and RSA (indirect effect = −0.107; 95% CI: −0.1653, −0.0566); SM played a mediating role between PASS and RSA, and the mediating effect was −0.068 (95% CI: −0.1166, −0.0308). SMT played a mediating role between PASS and PEE (indirect effect = −0.137; 95% CI: −0.1914, −0.0824); SM played a mediating role between PASS and PEE, and the mediating effect was −0.098 (95% CI: −0.1436, −0.0599). SMT played a mediating role between PASS and DSP (indirect effect = −0.051; 95% CI: −0.1013, −0.0092); SM played a mediating role between PASS and DSP, and the mediating effect was −0.141 (95% CI: −0.1945, −0.0942).

**TABLE 5:** Summary of the mediating effect of mental toughness and sports motivation between social support and athlete burnout.

**Effect**	**SC**	**Bootstrap *SE***	**95% CI**	**RME (%)**
**Overall athlete burnout**				
Total effect	−0.398	0.039	[−0.3699, −0.2184]	100
Direct effect	−0.150	0.036	[−0.1824, −0.0397]	37.69
Indirect effect	−0.248	0.032	[−0.3111, −0.1860]	62.31
PASS → SMT → AB	−0.113	0.028	[−0.1703, −0.0631]	28.39
PASS → SM → AB	−0.124	0.024	[−0.1751, −0.0793]	31.16
PASS → SMT → SM → AB	−0.011	0.008	[−0.0269, 0.0037]	2.76
**Reduced sense of accomplishment**				
Total effect	−0.326	0.012	[−0.0992, −0.0508]	100
Direct effect	−0.145	0.013	[−0.0582, −0.0087]	44.48
Indirect effect	−0.181	0.033	[−0.2517, −0.1195]	55.52
PASS → SMT → RSA	−0.107	0.027	[−0.1653, −0.0566]	32.82
PASS → SM → RSA	−0.068	0.023	[−0.1166, −0.0308]	20.86
PASS → SMT → SM → RSA	−0.006	0.005	[−0.0171, 0.0015]	1.84
**Physical and emotional exhaustion**				
Total effect	−0.374	0.016	[−0.1465, −0.0831]	100
Direct effect	−0.130	0.015	[−0.0694, −0.0103]	34.76
Indirect effect	−0.244	0.033	[−0.3080, −0.1808]	65.24
PASS → SMT → PEE	−0.137	0.029	[−0.1914, −0.0824]	36.63
PASS → SM → PEE	−0.098	0.021	[−0.1436, −0.0599]	26.20
PASS → SMT → SM → PEE	−0.009	0.006	[−0.0219, 0.0029]	2.41
**Devaluation of sports practice**				
Total effect	−0.320	0.018	[−0.1387, −0.0700]	100
Direct effect	−0.116	0.018	[−0.0723, −0.0032]	36.25
Indirect effect	−0.204	0.030	[−0.2661, −0.1476]	63.75
PASS → SMT → DSP	−0.051	0.023	[−0.1013, −0.0092]	15.94
PASS → SM → DSP	−0.141	0.026	[−0.1945, −0.0942]	44.06
PASS → SMT → SM → DSP	0.012	0.008	[−0.0295, 0.0041]	3.75

*PASS, social support; SMT, mental toughness; SM, sports motivation; AB, athlete burnout; RSA, reduced sense of accomplishment; PEE, physical and emotional exhaustion; DSP, devaluation of sports practice; SE, standard error; SC, standardized coefficient; CI, confidence interval; RME, relative mediation effect.*

## Discussion

### The Impact of Social Support on Athlete Burnout

The cognitive-affective stress model emphasizes that the cognitive assessment of the situation is one of the causes of mental fatigue and mental exhaustion. This scenario includes the athlete’s environment and the influence of environmental factors on it (Smith, 1986). Social support, as an essential social–environmental resource that athletes can obtain from coaches, family, teammates, and the organization, is an essential resource for coping with external pressure (Fisher, 1985). Nowadays, under the nation-level planned system, China continuously improves the system of on-the-job training for coaches based on the people-oriented principle, attaches great importance to the athletes’ benefits (Personnel Department of General Administration of Sport of China, 2019), and provides athletes with competition, training, and living expenses (Wang et al., 2014). All these promote athletes to obtain more resources coping with stressful situations and then relieve or keep from athlete burnout. A survey on 163 elite athletes from 14 sport types by Zhou and Guo (2007) pointed out that athletes’ mental health and perceived social support are essential predictors to sports fatigue. Their findings verified that social support can negatively affect athlete burnout (total effect = −0.398; 95% CI: −0.3699, −0.2184), and this outcome is also valid on weightlifters alone. Thus, hypothesis I is established.

### The Mediating Role of Mental Toughness Between Social Support and Athlete Burnout

The literature on athlete burnout paid little attention to the association between environment and personality (Gustafsson et al., 2011) and showed that social support had an impact on athletes’ mental toughness. Crust and Clough (2011) suggested that social support played an important role on the formation of athletes’ mental toughness, enabling them to challenge difficulties and adversities and enhance their self-confidence constancy. The improvement in social support level means that athletes can get various forms of resources (i.e., care, trust, respect, and advice) from teammates, coaches, or family, and all these can help athletes develop a strong sense of belonging (Ding et al., 2016), provide a variety of coping strategies (Shen, 2015), and rebound from adversity, therefore, enhancing psychological resilience. The present study confirmed that athletes’ social support could positively affect their level of mental toughness (beta = 0.310, *P* < 0.01). In addition, mental toughness could have a negative effect on athlete burnout (beta = −0.363, *P* < 0.01). As an essential psychological advantage, higher mental toughness level enables athletes to experience less physical and mental discomforts (Nicholls et al., 2008). Thus, athletes with high mental toughness have firmer beliefs against stressors and regard stressful situations as challenges rather than threats. This positive cognitive appraisal tendency can reduce stress perception, thereby suppressing burnout symptoms (Kaiseler et al., 2009). To sum up, we can see that social support can positively affect athletes’ mental toughness and thereby alleviate athlete burnout. That is, mental toughness plays a mediating role between social support and athlete burnout. Thus, hypothesis II is established.

### The Mediating Role of Sports Motivation Between Social Support and Athlete Burnout

Generally speaking, the factors affecting the intensity and direction of motivation originate from one’s internal needs and external conditions (Huang et al., 2018). The external conditions refer to environmental factors, that is, various stimuli outside the individual, including various biological and social aspects. For example, an athlete who participates in sports and desires success likes to get praise from his family and coaches or meet his own need of belonging by participating in a sports team. Social support has various forms and contents: not only objective forms of tangible support or specific technical guidance but also subjective forms of emotional warmth and personality respect, which integrate a robust support system (Freeman et al., 2011) to help athletes gain a strong sense of belonging and identity (Ye et al., 2016), to devote themselves with full of enthusiasm, vitality, great self-confidence, and firm determination to sports. Vallerand and Losier (1999) stated that social factors such as coaches’ behavior are one of the motivational factors that promote athletes to participate in sports. This study once again confirmed that athletes’ social support could positively affect their level of sports motivation (beta = 0.349, *P* < 0.01). In addition, sports motivation could have a negative effect on athlete burnout (beta = −0.354, *P* < 0.01). However, the concept and classification of sports motivation have not yet reached an agreement. Vallerand (1997) divided motivation into three types: intrinsic motivation, extrinsic motivation, and amotivation. Zhang and Mao (2004) divided it into two parts: participation tendency and avoidance tendency. But no matter what kind of sports motivation, its relationship with athlete burnout has been verified (Cresswell and Eklund, 2005; Zhang et al., 2010). If athletes regard sports as enjoyment, they will continue to invest in enthusiasm and maintain their sports career; on the contrary, if they regard sports as burden or constraints, they will experience burnout and exhaustion and withdraw from sports. Whether the athlete regards it as enjoyment or restraint is closely related to sports motivation (Raedeke, 1997). Therefore, the decline of sports motivation can be regarded as one of the early symptoms of athlete burnout. It can be seen that more social support can help athletes improve their sports motivation and devote more enthusiasm to training and competition, thereby reducing the occurrence of burnout. That is, sports motivation plays a mediating role between social support and athlete burnout (relative mediation effect accounts for 31.16%). Thus, hypothesis III is established.

### The Serial Mediating Effect of Mental Toughness and Sports Motivation

Mental toughness does not have a significant impact on sports motivation (beta = 0.099, *P* > 0.05). In this study, the serial mediating role of weightlifters’ mental toughness and sports motivation between social support and athlete burnout did not hold. Therefore, hypothesis IV is rejected. It may be that athletes’ mental toughness varies along with different sports (Gucciardi, 2009).

In addition, this study found out that the mental toughness of weightlifters and sports motivation also played a mediating role on social support and each dimension of athlete burnout. However, Figures 2–4 show differences in path coefficients of social support, mental toughness, and sports motivation on different dimensions of athlete burnout. This also suggests that we should consider the different symptoms and unique causes of athlete burnout in different dimensions in future research.

### Implications

With the continuous development of competitive weightlifting, athletes’ training experience has extended, and the number of competitions has increased. Due to long-term physical and psychological pressures, athlete burnout has frequently occurred, and related research has been increasing. Although some studies consider social support, most of them focus on different projects as a whole and lack the internal mechanism research between social support and athlete burnout (Liu, 2012). This study confirmed the double mediating role of mental toughness and sports motivation between social support and athlete, providing ideas for further exploration of the alleviation mechanism of athlete burnout in the future.

The research further clarified the value of social support and reminded coaches to pay attention to perfect the athletes’ social support system. In addition, research studies also proposed social support interventions, such as using commonly used remarks in the form of affirmative praise (Stragier et al., 2018) and group counseling with the theme of improving social support (Fan, 1996) to improve athletes’ mental toughness and sports motivation level and relieve the occurrence of athlete burnout.

Bowe believed that to effectively improve the mental toughness of athletes, it is necessary to strengthen the training of six psychological skills in a psychological training plan: competition trait anxiety, psychological preparation skills, achievement motivation level, trait self-confidence, concentration, and leadership skills (Zeng and Liu, 2013). Zhou and Chen (2013) improved the mental toughness of Judo athletes through 4 weeks of mental skills training, and some scholars have suggested that attribution training and goal setting can effectively improve sports motivation (Huang et al., 2018). Determination of two intermediary factors of mental toughness and sports motivation also provides new ideas for the intervention of athlete burnout. Coaches can improve the efficiency of athlete burnout relief through comprehensive interventions on social support, mental toughness, and sports motivation.

### Limitations and Future Research Directions

Although this study clarified the relationship across social support, sports motivation, mental toughness, and athlete burnout in weightlifters and confirmed the mediating role of mental toughness and sports motivation, it did not rule out the existence of other intermediary variables. For example, Shen (2015) found in a study on athlete burnout of college basketball players that social support can have an indirect impact on mental fatigue through emotional and stress-coping methods. It can be seen that emotions and coping styles may also be mediating variables between social support and athletes, which need to be further explored.

## Conclusion

The social support of weightlifters can negatively predict athlete burnout, suggesting the inhibitory effect of social support on athlete burnout. Current research showed that social support can impact athlete burnout through three paths: First, it directly affects athlete burnout; second, it has a mediating effect *via* mental toughness; and third, it has a mediating effect *via* sports motivation. The mediating role of mental toughness and sports motivation on social support and athlete burnout is established. Clarification of the path of social support, mental toughness, and sports motivation on burnout could provide ideas for further exploration of the alleviation mechanism of athlete burnout in the future and provide guidance for coaches in athlete burnout intervention programs.

## Data Availability Statement

The original contributions generated for this study are included in the article/supplementary material, further inquiries can be directed to the corresponding author.

## Ethics Statement

The studies involving human participants were reviewed and approved by the Ethics Committee at the Chengdu Sports University following the Declaration of Helsinki. The participants provided written informed consent to participate in the study.

## Author Contributions

S-YY: conceptualization and resources. YS: methodology and software. YS and S-YY: analysis, data curation, and writing—original draft preparation. Both authors have read and agreed to the published version of the manuscript.

## Conflict of Interest

The authors declare that the research was conducted in the absence of any commercial or financial relationships that could be construed as a potential conflict of interest.
